# New classification of industrial robotic gripping systems for sustainable production

**DOI:** 10.1038/s41598-023-50673-5

**Published:** 2024-01-02

**Authors:** Vitalii Ivanov, Vladyslav Andrusyshyn, Ivan Pavlenko, Jan Pitel’, Vladimir Bulej

**Affiliations:** 1 Department of Manufacturing Engineering, Machines and Tools, Sumy State University, 2, Rymskogo-Korsakova St., 40007 Sumy, Ukraine; 2 Department of Automobile and Manufacturing Technologies, Technical University of Kosice, 1, Bayerova St., 080 01 Presov, Slovak Republic; 3 Department of Industrial Engineering and Informatics, Technical University of Kosice, 1, Bayerova St., 080 01 Presov, Slovak Republic; 4 Department of Computational Mechanics named after V. Martsynkovskyy, Sumy State University, 2, Rymskogo-Korsakova St., 40007 Sumy, Ukraine; 5 Department of Automation and Production Systems, University of Zilina, 8215/1, Univerzitna St., 010 08 Zilina, Slovak Republic

**Keywords:** Mechanical engineering, Energy infrastructure

## Abstract

Robotics is an overarching trend in modern high-tech production, contributing significantly to automation. They are used in various industries to perform multiple tasks, and their number is constantly growing. Robots interact with the production object with the help of gripping systems, which are an essential component of industrial robots and manipulators designed for reliable grasping. Therefore, the process of design and rational selection of grippers for considering production conditions receives considerable attention worldwide. The article offers a comprehensive approach to the design of gripper systems as an integral element of the “gripping system – part – environment – production equipment” system to ensure further rational selection considering specific production conditions. A scientific approach to assessing the design of gripping systems was proposed to systematize knowledge in designing gripping systems. In the paper, the principal structural scheme of the robotic gripping system was developed, and the purpose of elements and design requirements were determined. Also, the sequence of stages in the process of selecting the elements of the gripping system has been proposed. The comprehensive system “gripping system – part – environment – production equipment” has been identified considering the mutual influence of structural elements. This work may be helpful to engineers and researchers while designing new gripping systems or selecting the most suitable one from the database. It can improve the rational selection of the element base and the structure of the gripping system by systematizing the experience in the gripper system design. Moreover, due to modern trends in automation and digitalization, the presented classification and coding system for gripping systems can be used in Computer Aided Process Planning and Computer Aided Gripping Systems Design systems. It can help to realize the approach “from the part geometry to the gripping systems design”. Also, it will ensure the production planning stage’s effectiveness due to reducing the time for robotic gripping systems’ design and increasing production safety, flexibility, autonomy, and performance.

## Introduction

Robotics is a relevant topic for scientific research, as it is one of the fundamentals of Industry 4.0^1^. It is widely used in many human activity areas: engineering, education, space and ocean exploration, military affairs, medicine, etc.^2^.

The International Federation of Robotics classifies two types of robots – industrial and service robots^3^. Based on the International Organization for Standardization^4^, the industrial robot is an “automatically controlled, reprogrammable multipurpose manipulator programmable in three or more axes, which can be either fixed in place or mobile for use in industrial automation applications”. The same standard defines a service robot as a robot “that performs useful tasks for humans or equipment excluding industrial automation applications”.

**Figure 1:**
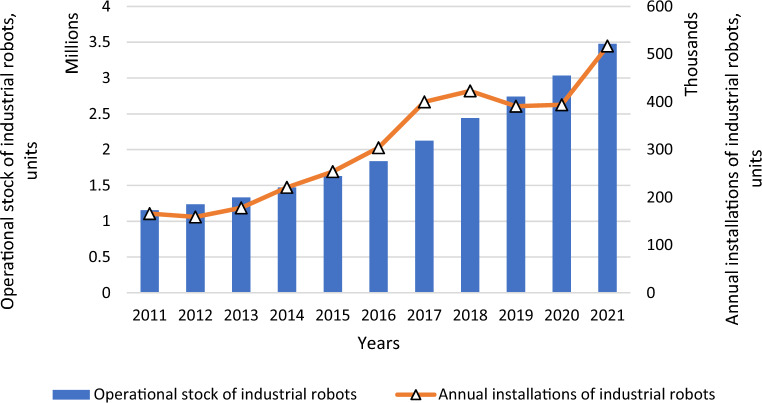
Dynamics of industrial robots worldwide in 2011–2021.

Based on the statistics^5^, 517,000 units of industrial robots and 121,000 units of professional service robots were installed in 2021. Consequently, sales of industrial robots are more than four times higher than sales of professional service robots, making research on industrial robots more relevant and a priority. Also, almost 3.5 million industrial robots were operated in factories worldwide as of 2021. This fact is confirmed by the dynamics of industrial robots (Fig. 1). Over the past five years, their number has increased by 89%. Notably, their demand will definitely grow in the future. Forecast data evidence that the need for new industrial robots will increase to 690,000 units in 2025. It should be noted that the number of collaborative robots is rising. In 2021, their number increased by 50% to 39,000 units.

**Figure 2:**
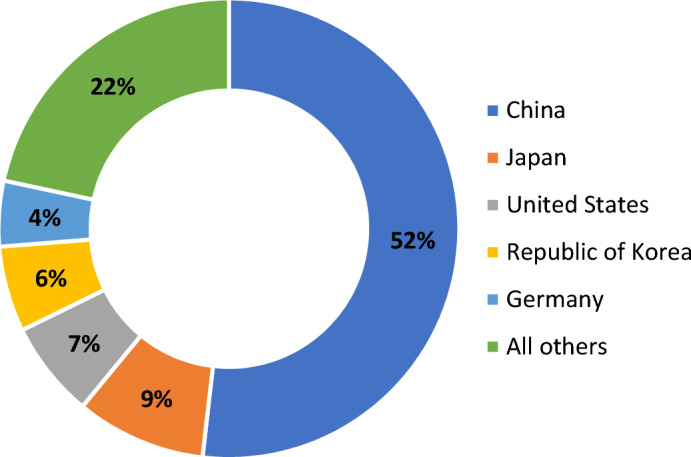
Distribution of industrial robots’ annual installations by country in 2021.

The most advanced technologies in developing and manufacturing robotic systems are concentrated in 5 countries – China, Japan, the United States, the Republic of Korea, and Germany and make up 78% of the annual installations worldwide. It is worth noting that China installed 268,200 units of industrial robots, which equals 52% of all installed industrial robots worldwide (Fig. 2). Production growth in China compared to 2020 is 51%.

According to the official statistics, in 2021, the average robot density in the manufacturing industry was 141 robots per 10,000 employees. Asia’s average robot density has grown by 18% since 2016 to 156 units per 10,000 employees. At the same time, European’s and America’s average robot density had been growing by 8% and achieved 129 and 117 robots per 10,000 employers, accordingly^6^.

**Figure 3:**
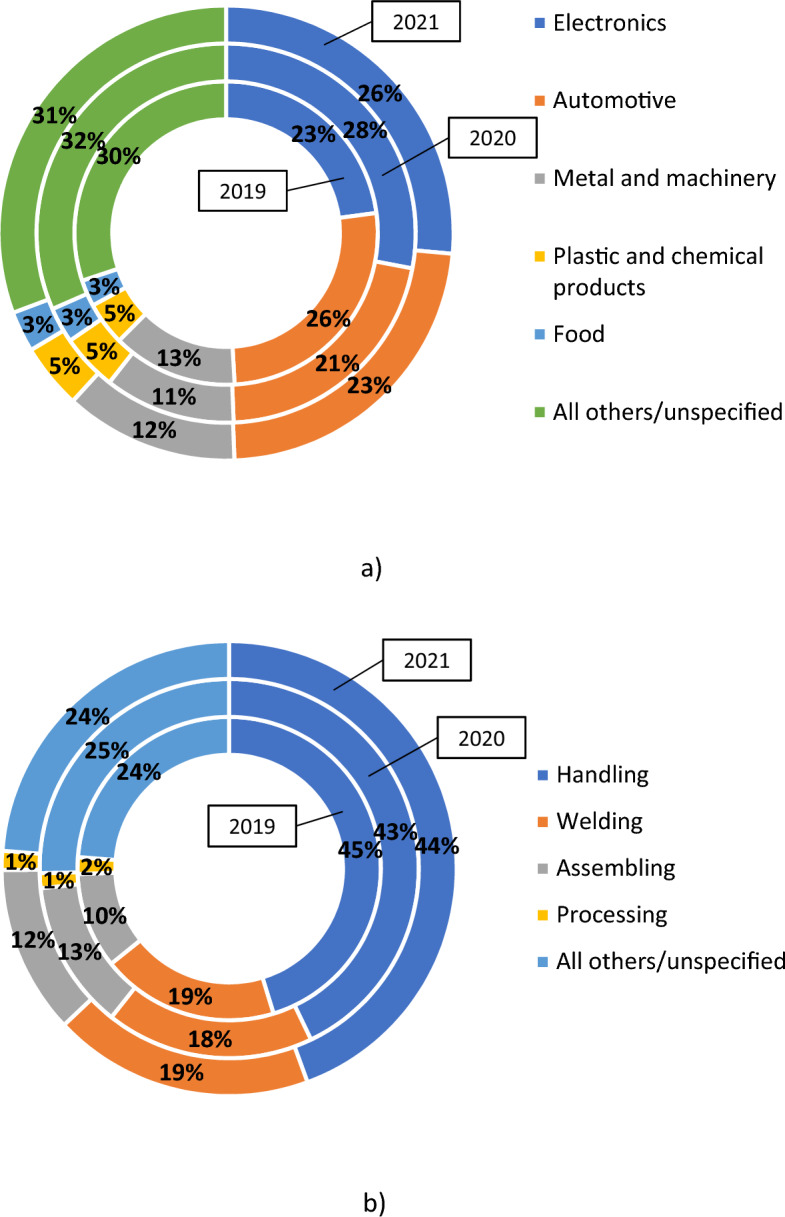
Distribution of industrial robots’ annual installations worldwide by customer industry (**a**) and by application (**b**) in 2019-2021.

Industrial robots are developed for different industries. The most in-demand industrial robots are for electronics, automotive, metal and machinery, plastic and chemical products, food, and others. Comparing the last two years, there has been an increase in the number of robots in the leading industries, particularly in electronics (+24%), automotive (+42%), metal and machinery (+45%), etc. Although, in the total volume of industries, the distribution remains almost unchanged during 2019–2021 (Fig. 3a). Progress in automotive and metal and machinery industries can be explained by the recovery of industries after COVID-19 and increased demand among customers.

Industrial robots are used for different applications, and the list of applications that an industrial robot can perform is only growing over time due to achievements in robotics. Handling remains the primary application in the industry. In 2021, annual installations of industrial robots for handling achieved 230,000 units and increased the previous year’s indicator by 36%. The share of handling among other applications exceeds 44% (Fig. 3b). A comparative analysis for the period of 2019-2021 evidenced that the order and distribution of applications of industrial robots are almost unchanged.

Industrial robots, in most cases, need end effectors to complete their tasks. The popularity of using robots for handling is also confirmed by the fact that popular CNC machine manufacturers provide solutions for their interaction (e.g. Mazak TA series, DMG MORI Robo2Go). Main applications such as handling and assembling (56% of the annual installations of industrial robots as of 2021) require robotic gripping systems as end effectors. By gripping systems, the authors mean end effectors, designed not only for seizing and holding but which are more complex in design compared to classical grippers and which can interact with the environment. Also, this terminology is already used by various companies that produce end effectors for industrial robots (e.g. Schunk, Schmalz, Gimatic, etc.).

Moreover, in addition to industrial robots, gripping systems are needed for manipulators that are built into the CNC machines (e.g. Okuma Armroid, Haas Mill Auto Parts Loader). It increases the area of use of the gripping systems. A number of manufacturers are involved in robotic gripper design. The distribution of grippers by the manufacturer was presented in the study^7^.

Currently, engineers are designing the gripping systems manually. It is a time-consuming process: according to Ref.^8^, the conceptual design of the gripping system alone can take about 30 man-weeks. Moreover, it causes increased material costs and errors due to the human factor. There is a relationship between the quality of the final product and the engineer’s experience.

Due to the focus of modern factories on automation and digitalization, the need to automate the design of gripping systems has arisen, and various researchers are actively working in this direction. The authors^8^ developed an approach for the automated design of gripping systems for manipulating and fixing parts from sheet metal for welding the cab body. The need to develop this approach arose due to regular changes in metal sheet shapes due to market requirements and the difficulty in applying flexible solutions due to strict accuracy and mass requirements.

However, the most popular work is design automation and optimizing fingers for gripping systems since they directly interact with the part, and their optimal choice significantly impacts the grasp’s quality. For example, the authors^9^ presented a soft finger drive design optimization model using an artificial neural network. Although the proposed model cannot create a soft finger from scratch, nevertheless, the proposed model saves money and time compared to the traditional trial and error optimization method. The authors^10^ have proposed a general method for optimized finger design for manipulating multiple objects. The proposed method can work with various scenarios of using gripping systems: for designing fingers for both shape and force grasp, internal or external grip, and one-sided or two-sided clamping.

Despite this, the topic of creating a universal and automated tool for designing a gripping system is still relevant^11^. In addition to design automation and optimization of elements of the gripping system, it is essential to consider its general structure for a wide range of applications. The authors^12^, when creating an approach to select the gripping system size automatically, also noted that a holistic study of the system is required for the possibility of automation.

Therefore, the following research gaps should be eliminated. Firstly, the gripping system’s structural elements are not considered an integral part of the system, and their roles are not clearly identified. Secondly, the sequence of the design stages in the process of selecting the elements of the gripping system should be considered depending on the production conditions. Finally, the gripping system design should reflect the mutual influence of structural elements in the comprehensive system “gripping system – part – environment – production equipment”.

The research aims to develop the classification of gripping systems for industrial robots by systematizing design experience, analyzing existing structures, and studies on flexible gripping systems ready for production in terms of the elements used in the design and justification of their choice. In addition, the technical documentation of the elements of gripping systems was analyzed. As a result, it will help make the design process of gripping systems more reasonable, reduce time waste during production planning, automate the production process, and implement intelligent production systems in individual production in the future.

The paper is organized as follows. First, in “Literature review” section, an overview of gripper design trends and how current realities have influenced the design of a modern gripper. “Research methodology” section shows the factors that influence the choice of the element base of the gripping device. “Results” section describes the classification of gripping devices, features, and the procedure for choosing the element base of the gripping system. “Discussion” section provides a gripper coding system, examples of its use, and examples of its use. Finally, conclusions are presented in “Conclusions” section.

## Literature review

**Figure 4:**
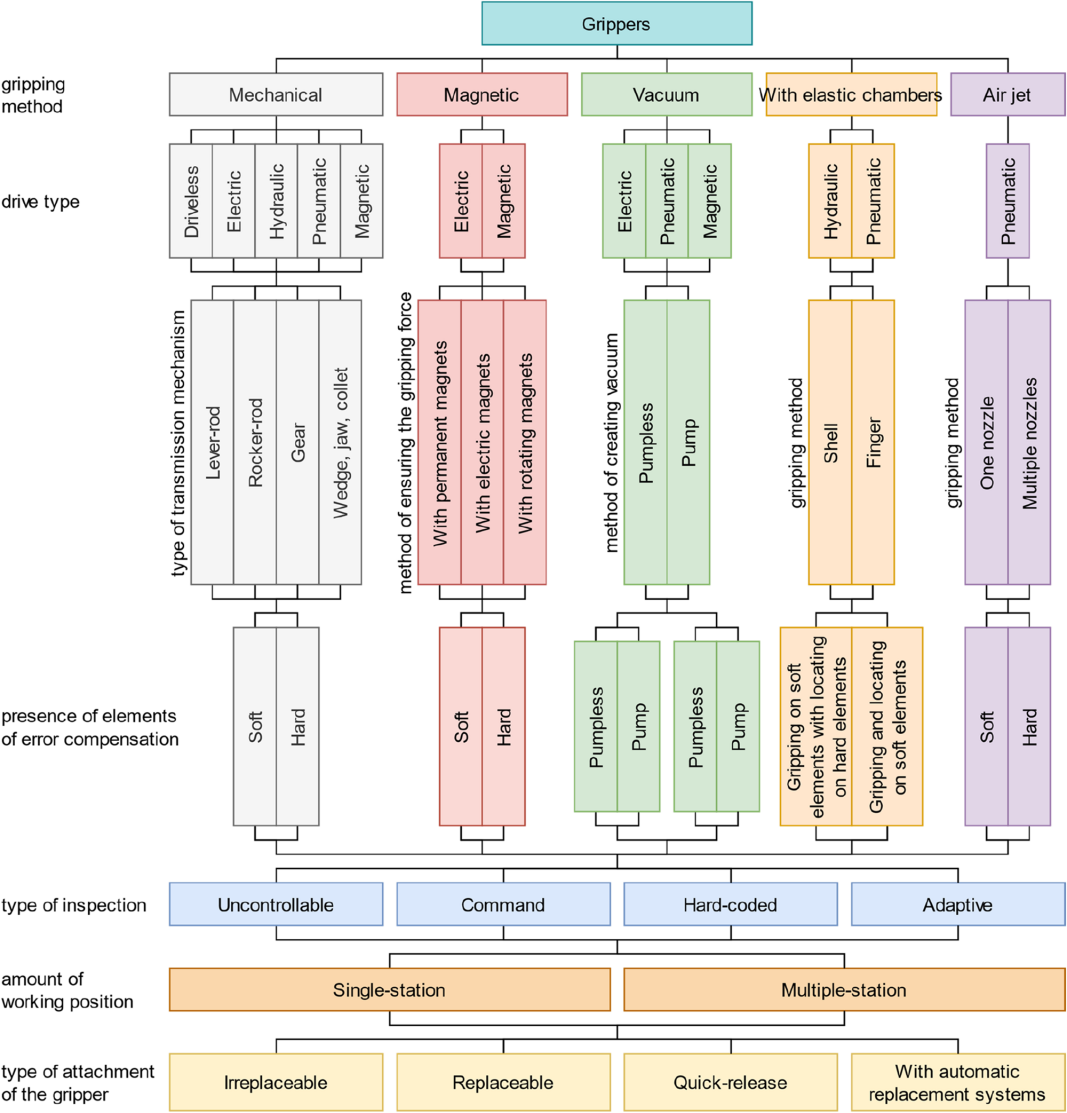
Classification of grippers.

For the starting point of this work, the authors take the classification of grippers^13^, which is shown in Fig. 4. Over a long period of design and application of gripping systems, a list of requirements for them has been developed as follows based on Ref.^13^: safety (the gripping system should securely hold the part both during operation and during an emergency stop); high accuracy (the gripping system must ensure repeatability of the position of the part relative to the connecting part); flexibility (the gripping system should grip a certain number of parts); ensuring the integrity of the part (the gripping system should prevent damage or destruction of the part); speed (elements of the gripping system should work as rapidly as possible); reliability (the gripping system should work out the number of cycles provided by designers); relative cheapness. These statements are also supported by the research study^14^. Moreover, an early basic universal classification of grippers is available.

Recently, the requirements for robotic cells and gripping systems have grown multiple. They should be safer due to the need to interact with people, must be able to interact with the environment following the requirements of Industry 4.0, and must also be flexible due to the transition of the industry to low-volume and single-unit production due to the need for an individual approach to each customer.

The first generation of industrial robots works only according to a particular program. It can interact with the environment to a limited extent, and a separate section is reserved for safety. Although industrial robots of the first generation perform most tasks efficiently, they have significant disadvantages: they cannot respond to errors in the production line and the impossibility of performing creative tasks (e.g. workpieces’ recognition). Thanks to discoveries in materials science and electronics, the rapid growth of computers’ computing power, and the development of data processing algorithms, it became possible to create a second generation of industrial robots. The second generation was created with these two disadvantages in mind: they can effectively interact with the environment.

Moreover, they can improve the efficiency of many tasks through the ability to interact with humans. They are also called collaborative robots, or cobots, and can interact with a person directly. For example, using collaborative robots for non-destructive disassembly can expand the range of components that can be disassembled and reused and make the process more cost-effective than destruction, which also positively impacts the environment^15^. Also, due to the introduction of artificial intelligence, they can perform simple creative tasks. The advantages mentioned above lead to increased sales of collaborative robots: in 2021, 39 thousand collaborative robots were installed, 50% more than in 2020 and 255% more than in 2017^5^.

Since the robot must now be close to a person and not work in a particular place reserved for itself, safety requirements have increased repeatedly: it is required to exclude the possibility of injury to workers. It significantly impacts the design of the gripping systems since the person works with the part manipulated by the gripping system, and the gripping systems have the most significant inertial forces in most cases due to the maximum lever length. There are several strategies for ensuring worker safety: crash safety, active safety, and adaptive safety. The main objective of crash safety is power and force limitations. Active safety deals with timely detecting imminent collisions and stopping the operation in a controlled way. Force, contact, proximity sensors, and vision systems are often used for these tasks. Adaptive safety systems apply corrective actions that lead to collision avoidance without stopping the device’s operation^16^.

In the field of proactive safety, both machine vision systems and sensors are used. For example, the authors presented the results of developing a collaborative production cell for assembling Rzeppa homokinetic joints because manual assembly causes muscular pain to workers^17^. With force and torque control and tracking of the operator’s hand via a machine vision system, this cell is compliant with safety standards and can be certified. Machine vision systems and sensors can also be used for learning by demonstration^18^. This article discusses the task of co-manipulation - the robot supports the weight of the transported load while simultaneously responding to human guidance. The Schunk FTD-Delta SI-660-60 sensor reads the torque and force data on the end effector, which is used for interaction with a worker. A neural network further processes this data to recognize intentional interaction with workers. This application also uses an external tracking system to record the trajectory of the movement, which is used for training. In addition, demonstration training (which may require force and torque sensors) can positively impact operator mental health by reducing stress and anxiety by making robot interaction and programming intuitive^19^. Machine vision can also be used to identify and locate parts, as in Ref.^20^, which made it possible to automate the riveting process, thereby increasing the efficiency of riveting compared to manual operation.

Moreover, a review of developments in machine vision showed that these systems could be used for various tasks due to their high flexibility^21^. They are also gaining popularity due to their accessibility and ease of setup. However, simultaneously, the use of machine vision systems in industrial applications is limited. During the assessment of the current level of readiness of security systems based on machine vision systems^21^, the authors noted the problems with the sensitivity of these systems to light and dust in industrial environments and difficulties when there are multiple people or large objects in the robot work cell. In addition, there are also issues with the presence of blind zones (obstructions)^22^. As mentioned, in most cases, researchers use commercial off-the-shelf solutions in the hardware part. For example, Schunk FT series or IPR F6D series sensors can be used to measure force and torque. Industrial cameras are used to implement machine vision. For example, companies Baumer (EX, CX, LX, AX, QX series), Stemmer Imaging, The Imaging Source (2, 27, 23 series), etc., produce industrial cameras.

As an example of active safety systems^23^, mentioned that a decoupling unit had been developed as an example of research in force reduction in the collision, which limits the force on the end effector during physical contact. The decoupling unit is implemented for the application with the screwing tool. It is designed according to functional safety requirements, but the concept for the balanced decoupling unit is not limited to this application. There are commercial versions of such units manufactured by Schunk (OPS and OPR series), IPR (ULD and ULS series), Zimmer (CSR and CRR series), etc. Still, they are more focused on protecting equipment and parts from possible damage and do not provide for use in collaborative systems, which is their disadvantage. But it is possible to implement this functionality since factory solutions can change the sensitivity during operation.

The direction of developing soft and sensitive skins for robots is also promising since they do not significantly affect the payload. Also, it will be possible to cover the robot’s entire body, including the end effector, using the skin. For example, the authors^24^ have developed sensitive skin for a robot with damping elements that can change the stiffness. Testing this solution in practice showed that the proposed skin is more sensitive than the solution of the collaborative robot from the factory.

Also, soft grippers can be partially attributed to systems that ensure collision safety. However, the main purpose of their development was an attempt to increase the flexibility and versatility of grippers while reducing weight. They have the following advantages compared to classic gripping systems, consisting of mostly rigid joints and links: ease of control and design, lightness, and versatility^25^. A study^26^ has shown that some soft gripper designs are effective and can be applied in real-world applications. In addition, work is underway to simplify the production of soft grips. Thus, the study’s authors^27^ designed a soft gripper with a high payload-to-weight ratio. The authors tested the gripper for durability and fatigue characteristics. The cheapness of the equipment required to manufacture this gripper, namely a low-cost 3D printer, makes this method of obtaining grips affordable.

Soft grippers are promising, but at the moment, they are used in mechanical engineering to a limited extent due to the low resistance of soft materials to chips, sharp edges, etc. However, they are actively used in other areas of production where the risk of mechanical damage is less, for example, in the food industry.

It should be noted that in practice, it is typical to apply active and proactive methods together to achieve an acceptable level of safety and performance. Active methods cannot fully interact with the environment. They cannot provide information that could allow recalculating the robot’s trajectory, allowing it to reach a given point safely. At the same time, proactive methods have a delay, and their effectiveness depends on the state of the environment.

Researchers are also working to improve the flexibility and performance of gripping systems to improve safety. The authors^28^ are focused on a new reconfigurable gripper design with three soft tactile fingers. A neural network processes the data received from the tactile fingers. Based on their readings in real-time, the grasping method is optimized to hold the object more reliably. A gripping system was also designed to increase the speed of working with parts fed randomly by the conveyor for delta robots^29^. Experiments have shown a 32% reduction in cycle time. In the future, the authors plan to reduce the size and weight of the obtained gripping system and test it in production conditions.

In addition, the need to change the design of gripping systems claims Industry 4.0 concept. Sensors are essential in the key to the Industry 4.0 concept as they keep operators, equipment, and products safe^30^. Thus, the authors^31^ noted the importance of sensors in the concept of Industry 4.0 since other elements of the concept (Big Data, IoT, system integration, etc.) are based on their readings. In addition, the analysis of the application by using sensors will allow evaluating the effectiveness and transfer experience to other robots. Also, the use of sensors significantly simplifies equipment maintenance^32^. Lists of commonly used sensors in manufacturing can be found in^33–38^.

## Research methodology

**Figure 5:**
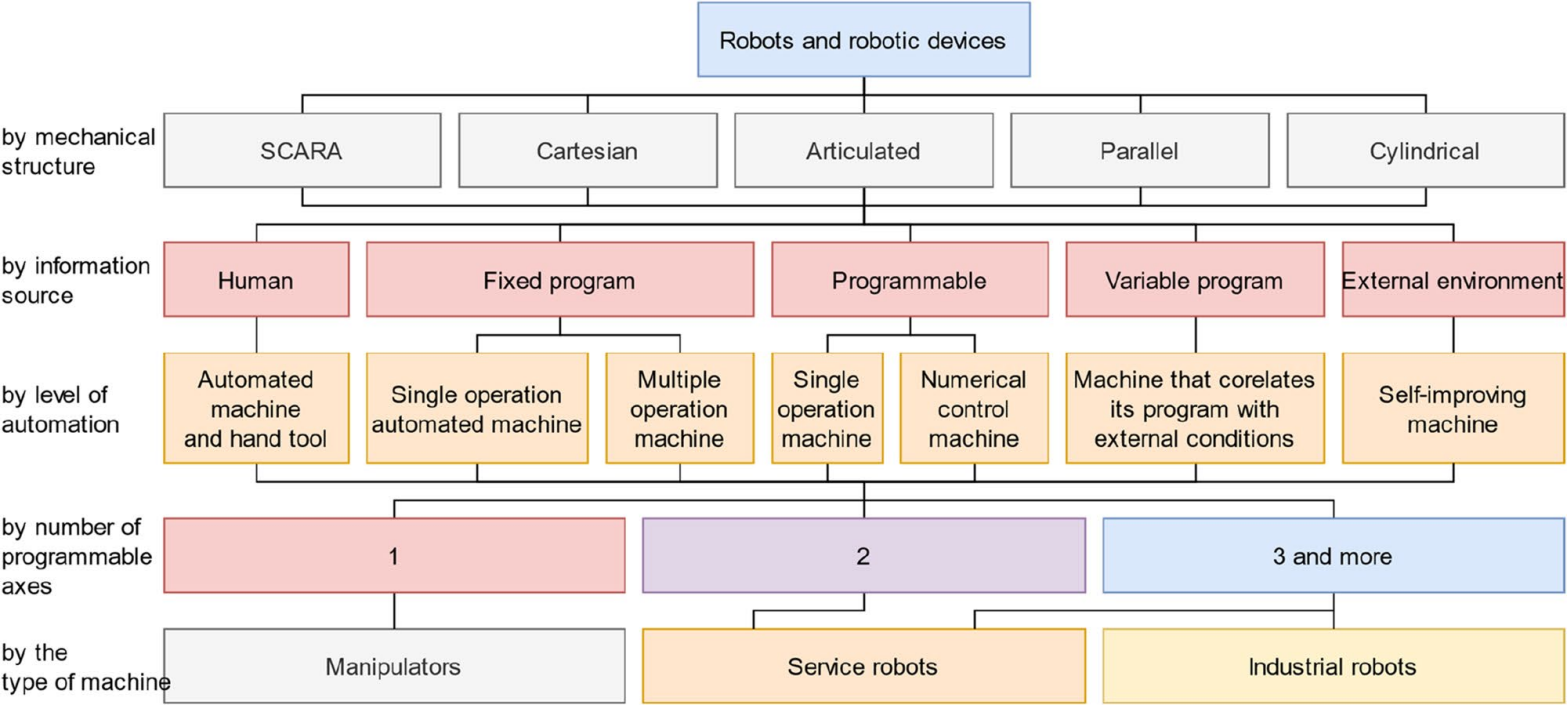
Classification of robots and robotic devices.

To understand what mechanisms can be related to robotic devices, it is necessary to provide a classification scheme for robots and robotic devices. The definitions from the standard^4^ were grouped by properties and eventually formed into the classification shown in Fig. 5. This classification has been supplemented with information about sources of information and levels of automation from^39^. Also, it should be noted that the following mechanisms are excluded from the classification because they are not related to the research topic: teleoperation facilities, automated guided vehicles, walking and crawling machinery, etc.

**Figure 6:**
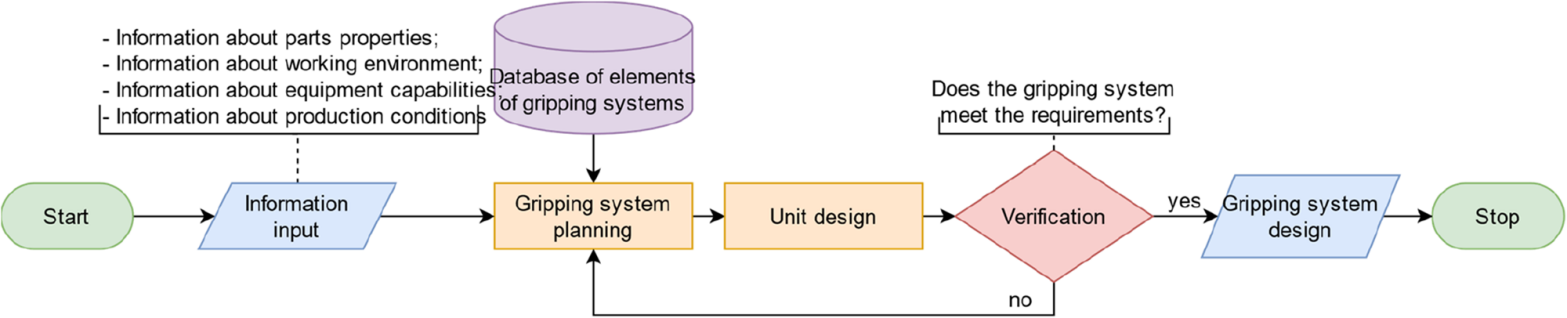
Flowchart of the gripping system design.

The design process consists of several stages, which are shown in Fig. 6. As input data, the gripping system planning stage accepts information about parts properties, the environment, the equipment of the robotic cell, and production conditions, as well as a database of grippers and sensors with their properties and features.

**Figure 7:**
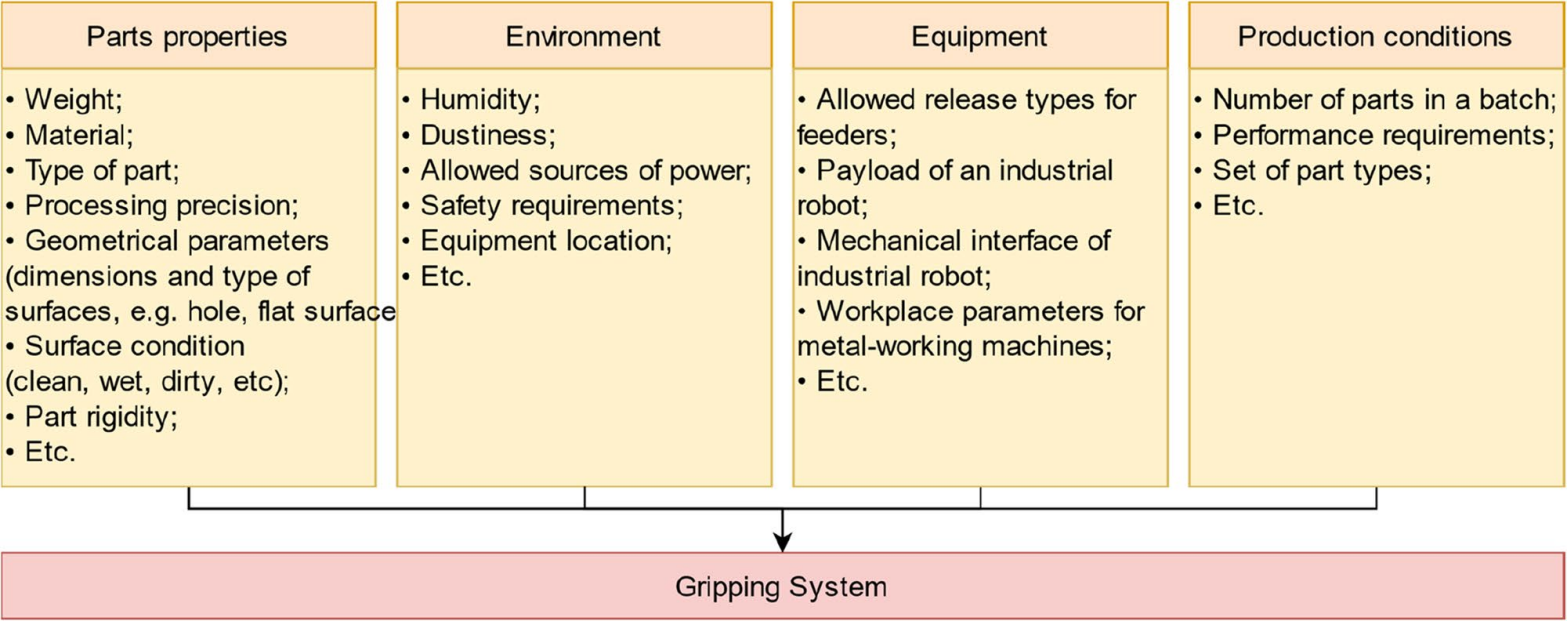
Flowchart of the gripping system design.

A detailed list of parameters that affect the gripping system is shown in Fig. 7. The output is an array of data about the elements of the future gripping system, sorted by the applicability of these elements in certain conditions. Further, this information is transferred to the block for the unit design, in which variants of the base plate, locating elements, fingers, etc., are generated based on the selected grippers and sensors. After that, the results are verified and checked for compliance with the requirements (safety, reliability, price, etc.). If the conditions are met, we have a bill of materials and design documentation at the output. Otherwise, it is necessary to revise the input data and conditions that apply to the design and start gripping system planning again.

The optimal choice of elements of the gripping system will determine its reliability and safety and the throughput of the robotic cell^40^. That is why it is necessary to have information about the features and limitations of the elements of the gripping systems. Classification of elements of gripping systems can help with this. With the increased number of variable elements of gripping systems due to the onset of Industry 4.0, it is necessary to revise the existing classification. This classification summarizes the experience in selecting elements of gripping systems depending on the input parameters. It can be used in the first stage of the computer-aided gripping system design.

## Results

**Figure 8:**
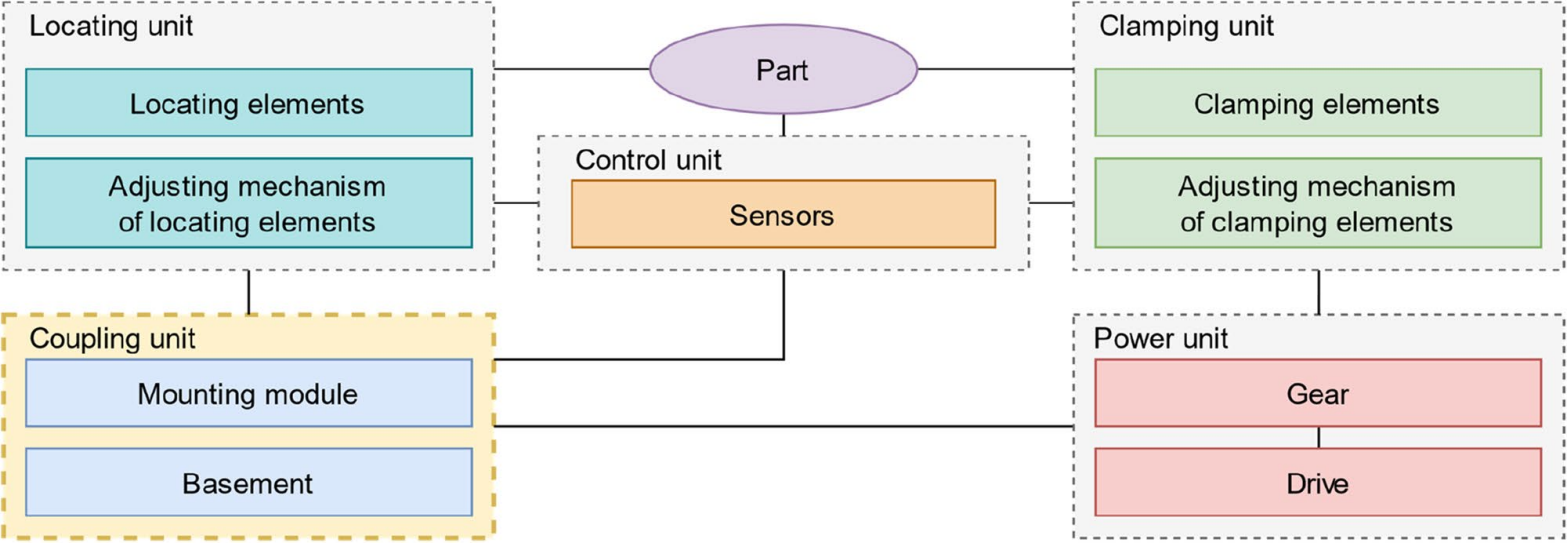
Structural diagram of a modern gripping system.

It should be noted there is no complete structure of the gripping systems. According to the authors, the gripping system should include five major units: coupling unit, power unit, clamping unit, locating unit, and control unit. The structural diagram of the gripping system is presented in Fig. 8. It describes the structural links between units.

It is necessary to consider the structure of units of gripping systems in more detail. Required units used in any gripping systems are the coupling unit, power unit, and clamping unit.

The power module is designed to create a force that guarantees a secure grasping of the manipulated part. Selecting elements of a power module is the first step in designing a gripper. Power modules by the gripping method are divided into impactive and astrictive gripping in mechanical engineering. Impactive gripping requires the motion of fingers to produce the necessary grasping force. Astrictive gripping is based on binding forces between surfaces^14^. The method of gripping is selected based on the parameters of the manipulated parts and the environment. Impactive gripping is a universal method and is suitable for most parts used in mechanical engineering. The module includes a drive and a gripping gear when using a power module with impactive gripping. In specific cases, transmissions consisting of several gears can be used instead of a single gear. Still, subsequently, this will increase in weight and a decrease in rigidity and reliability. In addition, when using a power module with impactive gripping, the presence of a clamping unit is necessary. The drives used in impactive gripping are electric, pneumatic, and hydraulic. The choice of the drive type depends on the availability of the required power sources, conditions of the environment, production requirements, part parameters, etc. The properties of different types of drives are given in Table 1^14^. Gears in gripping systems are used if necessary to separate, transfer force vectors, change their direction, or change the character of the movement (for example, from rotational to translational). In gripping systems, wheel, gear, cam, screw, wedge, rack and pinion, lever gears, and belt gears are most often used. The choice of gear depends on the required ratio and the need to split or redirect the power flow of the drive or if it is necessary to change the character of the movement. Also, it is popular among components manufacturers for gripping systems to produce drives and gears in one housing to reduce weight and overall dimensions.

**Table 1:** Properties of different drives.

Evaluation criteria	Electrical drive	Pneumatic drive	Hydraulic drive
Force-to-weight ratio	○	◐	●
Maintenance	◐	◐	◐
Controllability	●	○	◐
Insensitivity to dirt	◐	●	●
Emergency stop behavior	○	◐	◐
Costs	○	●	◐
Use in hazardous environments	○	●	○
Energy conversion efficiency	●	○	◐
Movement accuracy	●	○	◐

● – good; ◐ – acceptable; ○ – bad.

Astrictive grasping is narrowly focused and more efficient for working with flat parts such as metal sheets or glass sheets. This type of grasping is sensitive to the surface condition of the manipulated part. The drives used in gripping systems with astrictive grasping are magnetic and vacuum. The choice of type depends on the part’s material, the environmental conditions, and the available power sources. Magnetic gripping systems can only grasp magnetic parts. However, they are less sensitive to environmental conditions and easier to maintain. Both permanent magnets and electric magnets can be used in gripping magnetic systems. The advantages of permanent magnets are size and safety during an emergency stop, and the advantage of electromagnets is controllability.

Vacuum grippers can work with parts from different materials, but they are less resistant to wear. Also, they require equipment to create a vacuum, namely a vacuum generator, which entails additional costs. Vacuum generators can be either outside or on the gripping system in case of unique compact solutions. This feature should be mentioned to maintain compatibility between gripping systems in the robotic cell.

To increase the productivity of the production system, the gripper can be a multiple-station operation, which in turn requires the installation of a larger number of drives. Therefore, gripping systems can be classified into single-station and multiple-station operations by the number of working positions. In addition, when using several drives, in some cases, it is necessary to coordinate their work; for example, when in a gripping system, two pneumatic cylinders grasp a long shaft. That is why the drives can be consistent and inconsistent.

The clamping unit consists of clamping elements and a device for adjusting them. Impact gripping systems use fingers as clamping elements. Magnetic gripping systems use permanent and electric magnets. Vacuum gripping systems use suction cups. The fingers are divided into force-closure and form-closure according to the grasp type. Force-closure is a universal method, but its use is limited to handling soft, brittle, and delicate parts and parts with fine surfaces due to the principle of action. According to the type of shape, the force-closure fingers are flat, spherical, cylindrical, prismatic, or irregular. The choice of form mainly depends on the geometric parameters of the parts. By rigidity, fingers are hard or soft. Also, hard fingers can have a hard or soft coating surface. Parameters of the workpiece’s surfaces impact the choice of the type of fingers. The more delicate the part is, the softer the fingers are needed. Also, when using soft fingers or fingers with soft coating, the load capacity and durability of the grasping are reduced. In addition, the use of soft grippers is limited due to the reduced lifetime when there are sharp edges or chips on the part. In such cases, it is recommended to use form-closure fingers. They are less universal, as they require fitting to a specific part. To solve the problem of low flexibility of form-closure fingers, the authors created a technique for computer-aided design of fingers of this type for several parts^10^.

**Figure 9:**
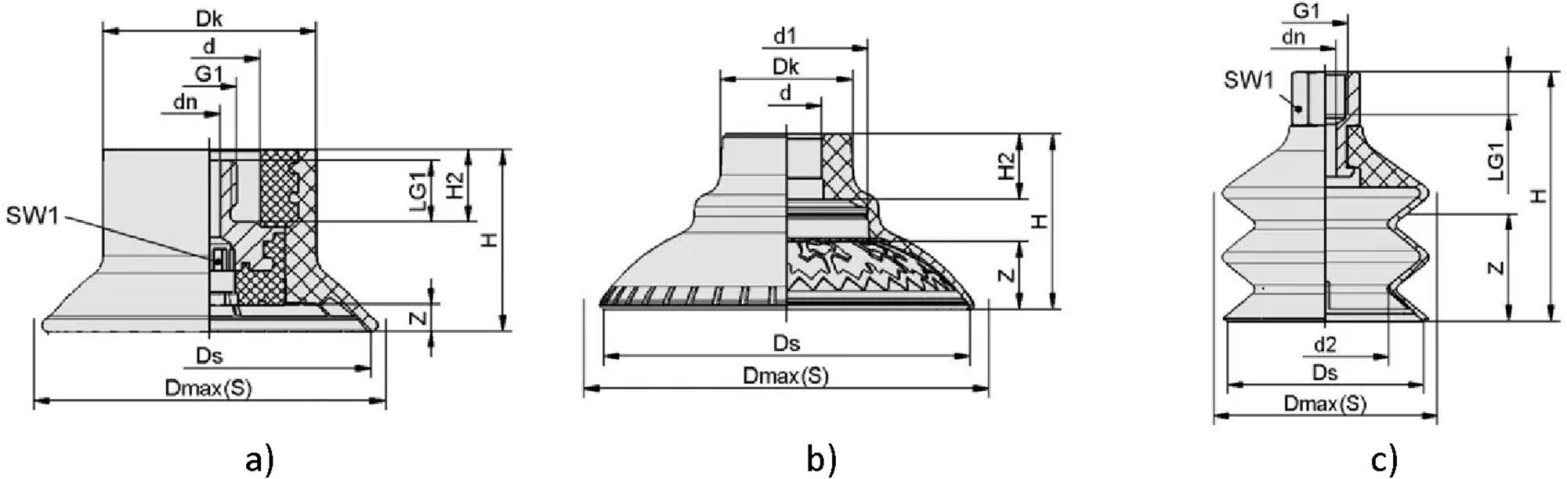
Types of suction cups: (**a**) - Flat suction cup Schmalz SAFT-C 60 NBR-60 M10-AG; (**b**) - Bell-shaped suction cup Schmalz SAXM 60 ED-85 SC055; (**c**) - Bellows suction cup Schmalz FSG 60 VU1-72 G1/4-IG ON.

Vacuum suction cups can be made in different designs using different materials, and the choice depends on the part’s parameters and production conditions. According to the design, suction cups used in mechanical engineering are divided into flat suction cups, bell-shaped suction cups, and bellows suction cups (Fig. 9). The features of flat suction cups include a higher gripping force with less deformation. This type is only suitable for grasping parts using flat surfaces with a slight curvature. Bellows suction cups are more effective for working with parts with large surface curvatures, such as car bodies, pipes, welded parts, etc. Bell-shaped suction cups are intermediate between flat suction cups and bellows suction cups. Also, suction cups are made in different shapes, and the most popular are round or oval. Round ones are universal, while oval ones are recommended for gripping thin and long workpieces, such as metal strips or frames. A proper selection of suction cup material is essential. The most popular are elastodur (ED), nitrile butadiene rubber (NBR), high-temperature rubber (HT), vulkollan (VU), and fluoroelastomer (FPM). Elastodur has excellent wear resistance and strength, good oil and weather resistance, does not affect paint adhesion, and works at temperatures from − 25 to + 80 C$^\circ$. Nitrile butadiene rubber has relative cheapness and good resistance to petroleum products, does not affect paint adhesion, and works at temperatures from − 10 to + 70 C$^\circ$. High-temperature rubber has good resistance to chemical attack, except for acids, wear resistance, and temperature resistance does not leave marks on the manipulated part, does not affect paint adhesion, and works at temperatures from − 10 to + 140 C$^\circ$. Vulkollan has excellent wear resistance and strength, good oil and weather resistance, and works at temperatures from − 40 to + 80 C$^\circ$. Fluoroelastomer possesses chemical resistance and does not leave marks on the manipulated part, and it works at temperatures from − 10 to + 200 C$^\circ$. Using other materials for suction cups in mechanical engineering is limited because of the influence on paint adhesion or the presence of traces after their usage^41^.

If necessary to design a flexible gripper, adjustment mechanisms are used. The adjustment can be carried out manually (without the drive) and automatically (with the drive) based on changeover time requirements. Manual readjustment is achieved by using additional structural elements. Automatic readjustment requires a drive and, if necessary, a gear. The description of the types of engines and gears made earlier is also valid for the adjustment mechanism. An example of using a device with automatic adjustment is given in work^29^.

A coupling unit is needed to set the position of all other units and connect them with the end joint of the industrial robot. This module is designed last, as its shape and composition depend on all elements’ geometric and operational parameters. The coupling unit includes a basement and mounting module. The basement is designed considering the load capacity of the industrial robot as well as the required accuracy and rigidity. Also, the basement can be made in the form of a body part of another module, for example, in RobotiQ solutions. Depending on the required gripping system change speed, the mounting module can be replaceable and quick-replaceable; quick-replaceable can be operated manually or automatically. Quickly replaceable systems are also used when there are many signal wires or pipes for compressed air.

In addition to the required ones, a locating or control unit can be installed to expand the technological capabilities of the gripping system. The locating unit is used to position the workpiece before clamping the part repeatably. The locating unit can have fingers of different shapes and an adjusting mechanism as the clamping unit.

The control unit consists of sensors. A sensor is a device that observes and measures a physical property of a natural phenomenon or man-made process and converts that measurement into a signal^42^. In gripping systems, sensors collect information about the state of elements of the gripping system, parameters of manipulated parts, or are responsible for interacting with the environment.

In the gripping system, sensors collect information about the state of units and parameters of the manipulated part and are also responsible for human-robot interaction. Angle and presence sensors are used to collect information about the state of the power unit. For pneumatic and hydraulic cylinders, inductive and magnetic sensors are most commonly used to determine the piston’s position. Optical and magnetic encoders are most used for electrical drives, but inductive and capacitive encoders are also used. Encoders convert the angular position of a shaft of electric drive to a signal. Information about the angular and linear position of the drive allows for indirectly determining the status of the gripper (open, closed, captured part, etc.). The choice of type of sensor depends mainly on the required accuracy and environmental conditions.

Consider the usage of sensors in the clamping unit in more detail. Force and presence sensors are used to collect information related to fingers. Force sensors can be divided into strain gauges, piezoelectric, optic, and resistive sensors. They are used on the fingers to control the gripping force to avoid the part falling out or being damaged. Presence sensors can be divided into inductive, magnetic, optic, capacitive sensors, and mechanical switches. Presence sensors are used to improve safety, for example, to directly confirm or deny the presence of a part in the gripping system. In the locating unit for locating elements, presence sensors (inductive, magnetic, optic, capacitive sensors, and mechanical switches) can be used to confirm the correct position of the part. For drives of adjusting mechanisms, angle and distance sensors can be used, which were described in the section with sensors for drives of the power module.

Presence, distance, image, and human-robot interaction sensors are used to collect information related to the coupling unit. In the mounting module, inductive sensors are used to determine the presence of contact between the gripping system and the industrial robot to improve the safety of the robotic cell.

Sensors in the basement are needed to control the parameters of parts, the state of units of the gripping device, and human-robot interaction. The following types of non-contact sensors are used in the basement to control the parameters of the part: distance and image sensors. Distance sensors, namely optical and ultrasonic sensors, are used, also as machine vision systems, to control the geometric parameters of the part. Image sensors, namely optical sensors and machine vision systems, are used to control the optical parameters of the part. To determine the correct position of the fingers or locating elements, the following distance sensors are used: optic, ultrasonic, magnetic, and inductive sensors. To check the parameters of human-robot interaction, resistive and capacitive sensors are used to detect direct contact with objects and tactile communication. For preliminary detection of contacts, camera vision systems, also as optical and ultrasonic sensors, are used.

When choosing a sensor, it is necessary to consider its parameters: sensitivity, stability, accuracy, speed of response, overload characteristics, hysteresis, operating live, costs, size, weight, stimulus range, resolution, selectivity, environment conditions, linearity, dead band output format, etc. Also, sensors can be classified into absolute and relative depending on the selected reference. Absolute sensors measure relative to an absolute physical scale, while relative sensors provide measurements that relate to the value taken as the initial one. Moreover, sensors can be contact or non-contact^43^. Sensor parameters mostly depend on the physical principle of operation and its implementation by the manufacturer.

**Figure 10:**
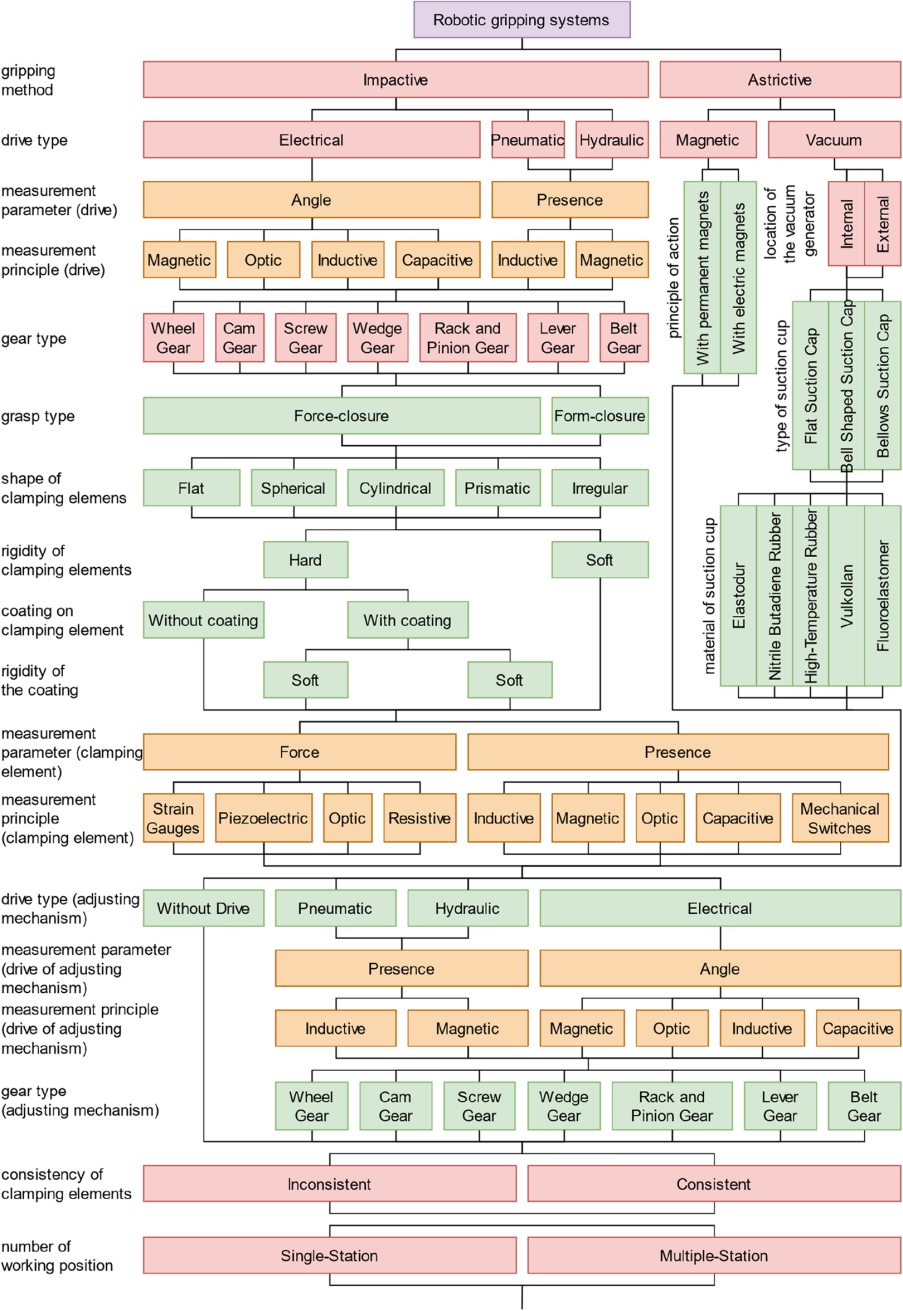

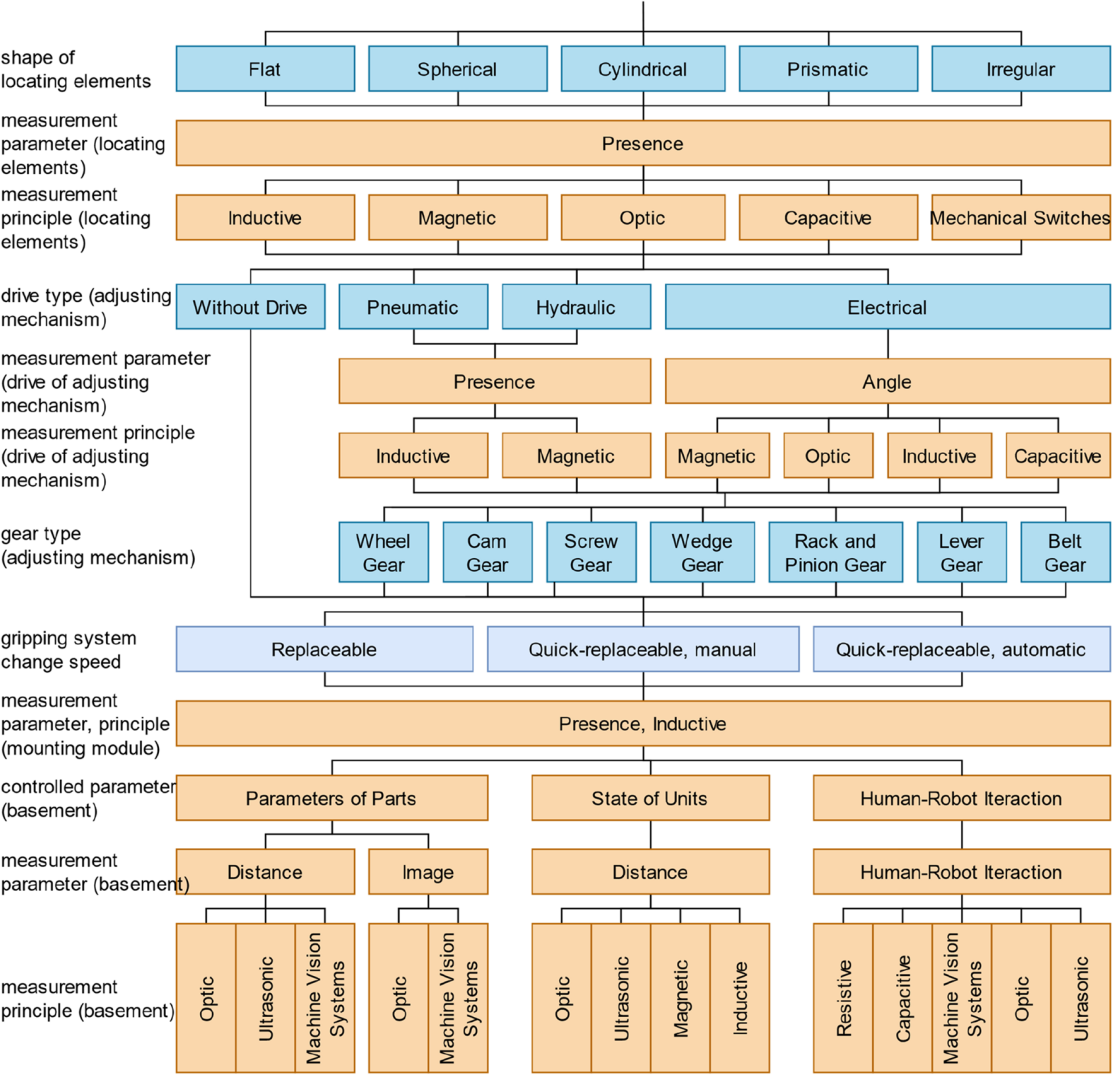
Classification of modern gripping systems.

As a result, the final list of the main elements of modern gripping systems can be put into a classification (Fig. 10). This classification can be used, for example, in CAD and CAPP systems, to describe the principal elements and properties of a gripping system. First, it is necessary to classify gripping systems according to the properties of the elements of the power and clamping units since these units directly interact with parts and respond to reliable grasping, which is the primary purpose of gripping devices. In addition, when choosing elements of the power and clamping modules, we limit the range of parts the gripping system can handle and introduce limitations on the production environment conditions. First, the gripping method is worth mentioning, as this parameter limits the type of parts the gripping system can work with, for example, general mechanical parts, flat parts, etc. With the impactive gripping method, the choice of the drive type follows since this parameter establishes limitations on the parameters of the manipulated parts and the production conditions and introduces requirements for the availability of power sources in the robotic cell. Further, suppose it is necessary to control the state of the drive. In that case, it is required to specify the measured parameter and then specify the measurement principle since it affects the sensors’ accuracy and reliability. The following is a description of the drive type since this affects the final stroke length of clamping elements, the force of their grasping, and their number. After that, we classify gripping systems according to the grasp type since this affects the design of clamping elements and limits the number of parts that the gripping system can grasp due to the geometric parameters of the parts. Next, it is necessary to mention the rigidity of clamping elements, the coating of their working surfaces, and the rigidity of the coating, as this affects the accuracy and strength of parts and their surface layer that can be clamped with this gripping system. After that, if it is necessary to control the interaction between clamping elements and the part, it is essential to specify the measured parameter and then specify the principle of measurement since this will have an impact on the list of parts that can interact with this gripping system, as well as on the design of the clamping elements.

With the astrictive gripping method, the drive type is also selected. For vacuum drives, it is also worth mentioning the location of the vacuum generator, as this will affect the final weight of the gripping system. When describing clamping elements with an astrictive gripping method for magnetic drives, it is worth specifying the principle of the magnet, as this will affect the logic of the control of the gripping system, and, when using a permanent magnet, an adjusting mechanism for a controlled gripping process may be required. When describing the clamping elements with the astrictive gripping method for vacuum drives, it is necessary to mention the type of suction cups, as this limits the shape of the part that is most optimally gripped by one or another suction cup. In addition, it is worth specifying the material of the suction cups, as the right choice of material for specific production conditions and tasks will ensure high productivity with high process reliability.

After that, it is worth mentioning the mechanism for adjusting the clamping elements, which is used if necessary to increase the flexibility of the gripping system, and which, in this case, acts as a link between clamping elements and the power unit. First, it is necessary to consider the choice of the drive since the method of changeover depends on this (manual or automatic), and the choice of the drive depends on the production conditions and introduces requirements for the availability of power supplies. Also, as in the power unit, it is necessary to mention the parameters and physical principles of the sensors that will control the state of the adjustment mechanism drives. In addition, it is worth noting the type of gear of the adjustment mechanism, if necessary to use it, to describe the magnitude and number of adjustments when using drives.

Next, it is necessary to mention the consistency of drives since when clamping complex parts, it may be necessary to additionally apply several coordinated drives with their own set of clamping elements and adjusting mechanisms. For example, multiple consistent drives would need to be applied to avoid deflection to manipulate long shafts with low stiffness. After that, it is worth describing the working positions of the gripping system because, to increase productivity, the gripping system can have several sets of power units with its own set of clamping elements and adjustment mechanisms for performing different tasks or for grasping different parts.

Next, it is worth describing the parameters of locating elements since they directly contact the part and affect the gripping system repeatability, which is essential for some applications. Further, suppose it is necessary to control the interaction of the locating elements and the part. In that case, it is required to specify the measured parameter and then specify the principle of measurement since this will affect the design of locating elements and the range of parts that can interact with this gripping system. In addition, locating elements and clamping elements can have an adjusting mechanism, which is also worth describing. The next thing to mention is the gripping system change speed, as this parameter will depend on the mechanism of changeover, and its choice will depend on the list of parts, production conditions, and the number of drives and sensors. In addition, it is necessary to consider the need to control the tool change mechanism. Therefore, it is required to note the measured parameter and the principle of sensors for monitoring the gripping system change mechanism.

As a result, for the possibility of designing the basement, it is necessary to mention additional sensors for interaction with the gripping system elements, the part, and the environment. In addition, it is essential to note their measured parameter and the principle of measurement to determine the capabilities of the gripping system.

**Figure 11:**
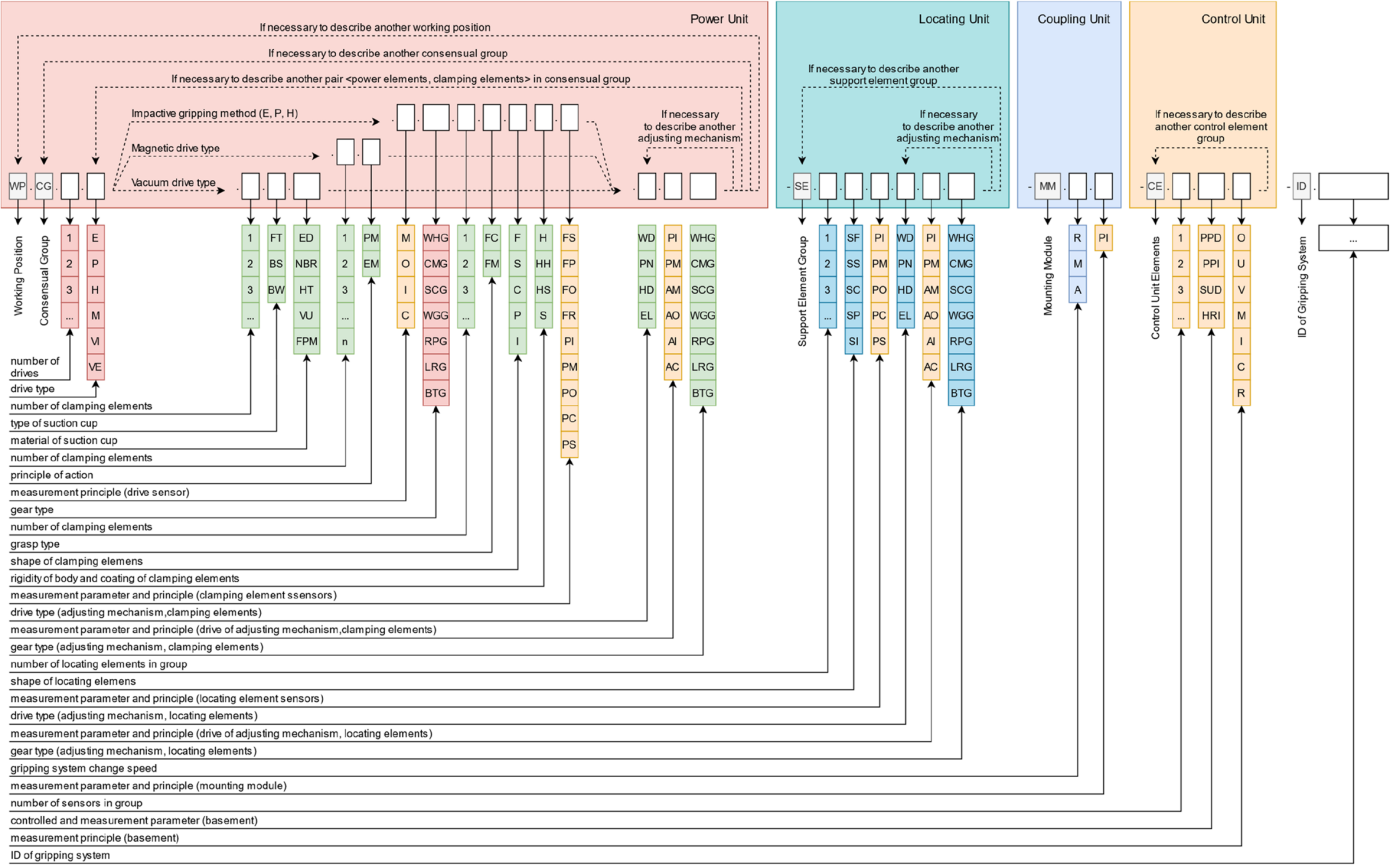
The gripping system’s coding system.

The proposed classification of modern gripping systems can be used as guidelines for the design of gripping systems. Also, the information described above can be entered into a coding system (Fig. 11) and used to select suitable grippers already available in the robotic area under the new part.

## Discussion

**Figure 12:**
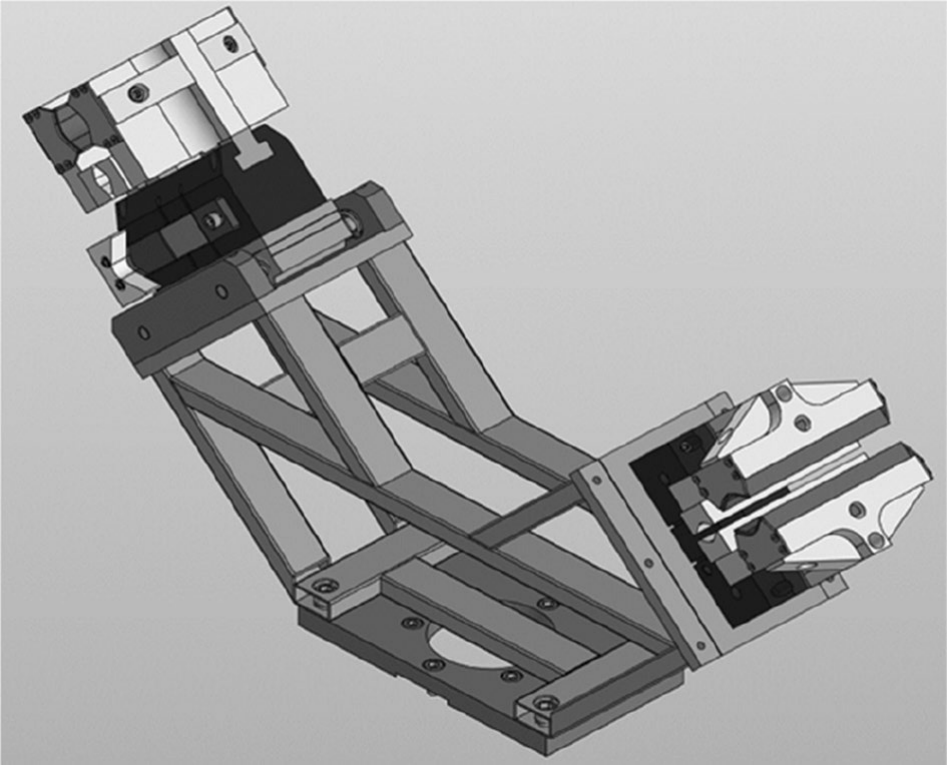
Gripping system for handling rotational parts^47^.

**Figure 13:**
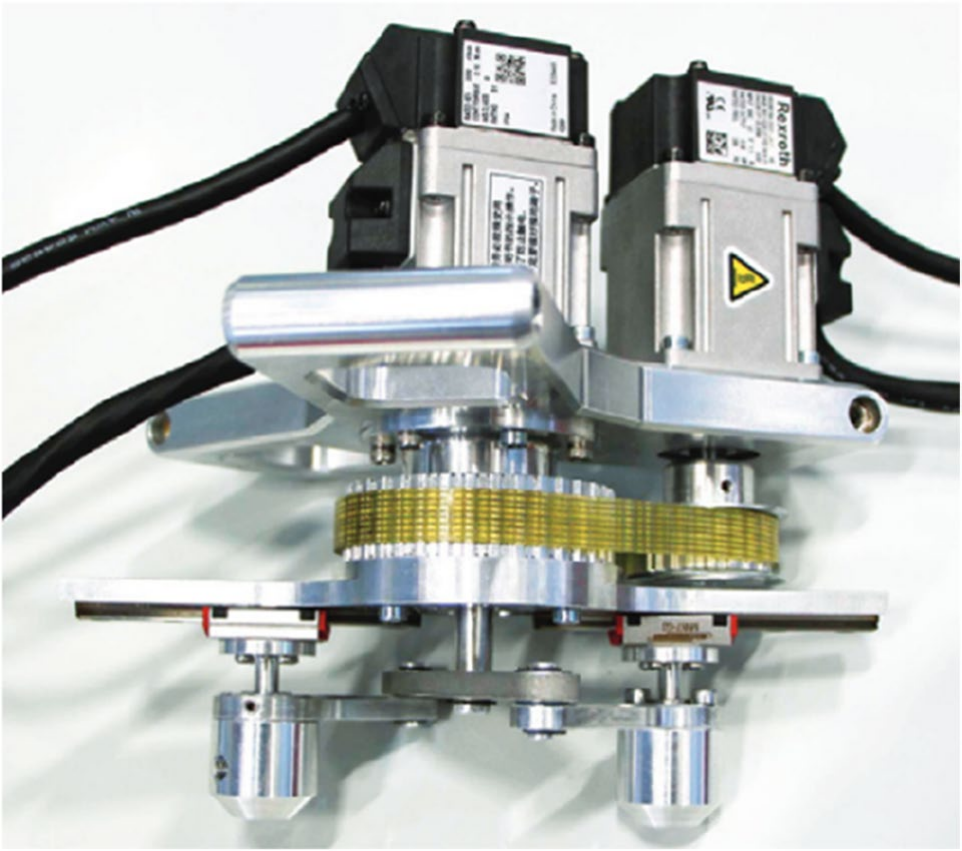
Gripping system for picking up two objects simultaneously^29^.

**Figure 14:**
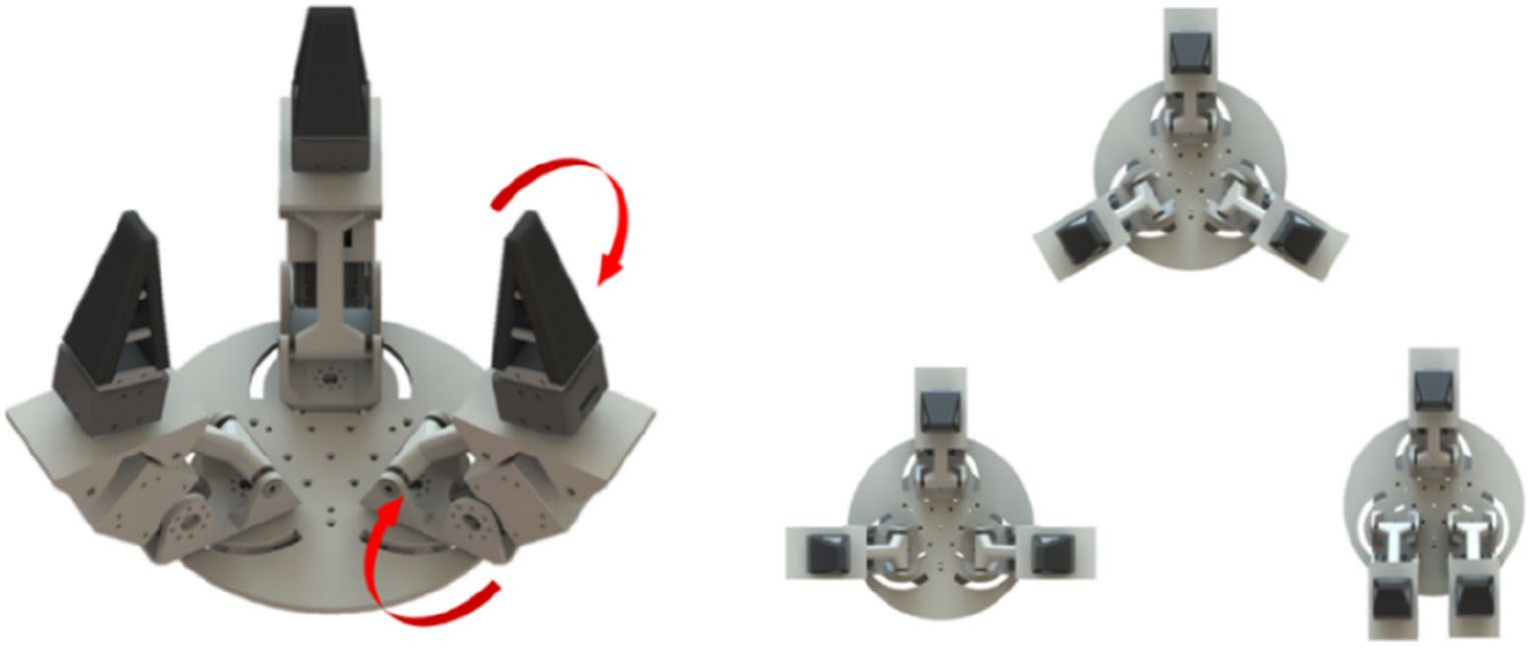
Gripping system for interactive grasping^28^.

The research^44^ provides an exhaustive classification of pneumatic grippers. The proposed classification contains a description of the properties of the elements of pneumatic gripping systems, as well as examples of the design of pneumatic gripping systems. The authors also consider trends in the development of gripping systems and substantiate the need for scientific research in the design of gripping systems and the systematization of knowledge in this area. However, this article is limited to describing elements of power and clamping unit. Moreover, the classification of elements of the gripping systems proposed by the authors does not take into account the applicability of the considered elements in various industries, and the future use of the proposed classification is also not considered. The research study^14^ contains a section devoted to classifying grippers. It is only dedicated to the type of gripping method, i.e. it describes only the power unit. Article^45^ provides a brief classification of grippers based on their configuration and application but without delving into the structure of gripping systems and the description of the elements. The article^46^ also provides a classification of grippers, but only certain aspects of the power and clamping modules are covered there, i.e. it is not suitable for complex grippers. This article can be declared innovative since it affects more complex gripping systems and considers their internal structure. As an example, this classification system can be used for the following complex gripping system (Figs. 12, 13 and 14). The structural codes assigned based on the developed classification and the coding system are presented in Table 2.

**Table 2:** Structural codes for gripping systems.

Gripping system	Manufacturer	Structural code (without ID of the gripping system)
Figure 12	LLC CTI^47^	WP.CG.1.P.M.2.FC.P.HS. WP.CG.1.P.M.2.FC.P.HS-MM.R
Figure 13	T. Atakuru et al.^29^	WP.CG.1.VE.2.FT.ED.EL.AM.BLG.EL.AM.LRG-MM.R
Figure 14	L. Yang et al.^28^	WP.CG.3.E.1.FC.F.S.FO.EL-MM.R

It is worth mentioning that this coding system does not mention the parameters of the elements, which is why there is an ID of the gripping system at the end of the classification because it is necessary to inform about the parameters of the gripping system.

For example, the gripper system ID may contain information about such important parameters as prehension force, gripping range settings, closing and opening time, load limits, overall dimension, deadweight, etc. Moreover, additional information can be added, such as operational temperature, mass moment of inertia, reproducibility and accuracy, maintenance cycle, energy consumption, service life, etc.^14^.

Due to the focus of modern enterprises on automation and digitalization, the presented classification and coding system for gripping systems can be used in Computer Aided Process Planning and Computer Aided Gripping Systems Design systems. Previously designed grippers and the products manipulated by them can be represented using a structural code. As a result, the “gripping system - production” combination and the description of the production conditions can be filled into a database, which can later be the basis of a gripping systems design algorithm using machine learning. Moreover, the popularity and ease of setup of cloud computing and big data may facilitate the collection of the above correspondences on a global scale.

## Conclusions

A critical analysis of the current state of robotics, mainly an overview of the spread of robotics and robotics-based applications, the role of robotics in various industries, and current trends in the field of robotics have been made for grippers and gripping systems of industrial robots. The article highlights the role of gripping systems in modern robotic production cells, how robotics trends have influenced gripping systems and the current state of the market of gripping systems elements.

The problems with the systematization of knowledge in designing gripping devices have been identified. Based on this, the authors, for the first time, proposed a scientific approach to considering the design of gripping systems to systematize knowledge in the field of designing gripping systems, which contains the following aspects. The functions of structural elements of the gripping system, i.e. their role in the system, have been substantiated. The sequence of stages in the process of selecting the structural elements of the gripping device has been proposed. As a result, the comprehensive system “gripping system – part – environment – production equipment” has been identified, considering the mutual influence of structural elements.

Also, the proposed approach applies to classifying a broad class of gripping systems for assembly and handling using a new coding system. However, since this research deals with traditional industrial robot gripping systems widely used in mechanical engineering, it does not include construction types such as arms.

This work will be helpful to engineers and researchers while designing new gripping systems or selecting the most suitable one from the list already introduced into current production for manipulating new products. It can rationalize the selection of the element base and the structure of the gripper system by systematizing the experience in the field of gripper system design.

In the future, it is possible to develop a method for the automated design of gripping systems using artificial intelligence, aiming at implementation in industrial enterprises to overcome Industry 4.0 challenges. Notably, a database of elements of gripping systems and their manufacturers will be created, focusing on mechanical engineering applications.

## Data Availability

The data presented in this study are available on request from the corresponding author.
